# Adaptive Weighted Data Fusion for Line Structured Light and Photometric Stereo Measurement System

**DOI:** 10.3390/s24134187

**Published:** 2024-06-27

**Authors:** Jianxin Shi, Yuehua Li, Ziheng Zhang, Tiejun Li, Jingbo Zhou

**Affiliations:** School of Mechanical Engineering, Hebei University of Science and Technology, Shijiazhuang 050018, China; shijianxin197@163.com (J.S.); 18033857268@163.com (Z.Z.); 001036@hebust.edu.cn (T.L.)

**Keywords:** line structured light, photometric stereo, cooperative measurement, adaptive weighted data fusion

## Abstract

Line structured light (LSL) measurement systems can obtain high accuracy profiles, but the overall clarity relies greatly on the sampling interval of the scanning process. Photometric stereo (PS), on the other hand, is sensitive to tiny features but has poor geometrical accuracy. Cooperative measurement with these two methods is an effective way to ensure precision and clarity results. In this paper, an LSL-PS cooperative measurement system is brought out. The calibration methods used in the LSL and PS measurement system are given. Then, a data fusion algorithm with adaptive weights is proposed, where an error function that contains the 3D point cloud matching error and normal vector error is established. The weights, which are based on the angles of adjacent normal vectors, are also added to the error function. Afterward, the fusion results can be obtained by solving linear equations. From the experimental results, it can be seen that the proposed method has the advantages of both the LSL and PS methods. The 3D reconstruction results have the merits of high accuracy and high clarity.

## 1. Introduction

Line structured light (LSL) sensors have the advantages of simple structure, high accuracy, and low cost. A typical LSL sensor consists of a camera, a laser line projector, and a frame that connects them together [1,2]. Currently, they are widely used in quality evaluation [3], geometric measurement [4,5], visual tracking [6], railway inspection [7], etc. In the measuring process, a laser line is projected onto the object, and the camera captures the perturbed stripe image that carries the profile information. Camera coordinates of each point on this profile can be solved with camera intrinsic parameters and the laser plane equation [8]. 

Photometric stereo (PS) measurement has the advantages of fast measurement speed, simple structure, and high clarity. The classical PS system consists of a camera and several light spots [9]. It has been applied in defect detection [10,11,12,13,14], face recognition [15,16], and cultural heritage digitization [17]. The measurement process of PS is achieved by taking images of the object under different light spots. The surface normal vector of the object can be calculated according to the light and dark changes. The 3D result can be achieved by gradient integration [18,19].

LSL and PS are two measurement techniques that have the advantages of low cost, high degree of automation, and simple operation. Although LSL can provide 3D geometrical information with high accuracy, its clarity is highly affected by the noises introduced in center extraction of the laser stripe and the sampling interval of the scanning. On the contrary, PS is sensitive to the details on the object. The measurement accuracy is low due to the noise accumulation in gradient integration. Therefore, how to achieve high precision and high clarity results efficiently is a key issue in the research of 3D measurement. Cooperative measurement with LSL and PS may be a solution.

Based on the above considerations, Nehab et al. [20] fused the position information obtained from a depth scanner with the normal vectors computed by PS, combining the advantages of both measurements. Haque et al. [21] added Laplace smoothing terms to the optimized surface equations, aiming to make the result smoother at the edges, but the reconstructed surfaces had holes. Zhang et al. [22] constructed an optimized surface equation for data fusion where a Gaussian filter was designed by considering both the neighborhood and depth values, while it required a complex iterative process and was time consuming.

Okatani et al. [23] solved the optimization problem efficiently by using recurrent belief propagation. It has the limitation that accurate results can only be obtained when an appropriate confidence level is selected. Bruno et al. [24] proposed a method combining coded structured light and PS for the 3D reconstruction of underwater objects, but the image acquisition time is very long and further improvement is needed for practical applications. Massot [25] and Li [26] also combined structured light and PS for the 3D reconstruction of underwater objects. Riegler et al. [27] combined photometric loss and geometric loss to train a model in a self-supervised way, but the accuracy of their reconstruction results was not high. Lu et al. [28] proposed a multiresolution surface reconstruction scheme, which combines low-resolution geometric images with PS data, but the iterative process in their algorithm takes a long time. Li et al. [29] proposed a novel local feature descriptor to fuse neighborhood point cloud coordinates and normal vectors. The accuracy of the results is improved, but the computation time is long, especially when the number of point clouds is large. Antensteiner et al. [30] proposed a fusion method based on the total generalized variance to improve the accuracy, but its computational speed still needs to be improved. Hao et al. [31] corrected the deviation of the PS by fitting an error surface using a 3D point cloud of structured light. The depth of PS is achieved by the integration process, and the noises are also accumulated. 

In this paper, we propose an adaptive weighted fusion algorithm based on the angle of the adjacent normal vector. Firstly, the PS method is used to calculate the surface normal vectors, and perform weighted fusion by use of the normal vector angles of the neighboring points. Next, the error function of the fused surface is established, which consists of the error in the 3D point cloud and the normal vector. The fusion result can be obtained by establishing a sparse matrix and solved with a linear equation. Our algorithm has the advantages of both the LSL and PS methods, and can achieve a high accuracy and high clarity result.

## 2. Measurement Principle

**Figure 1:**
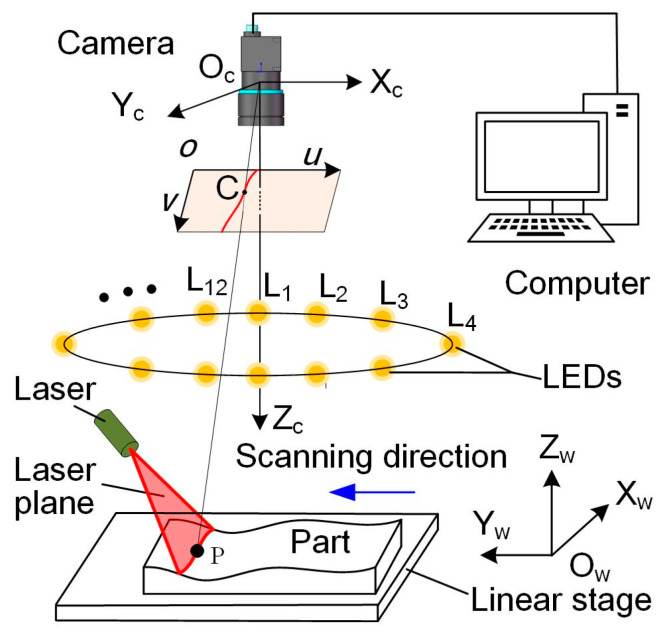
Illustration of the cooperative measurement system.

The LSL-PS system measurement principle is shown in Figure 1. The LSL sensor consists of a camera and a laser line projector. The laser plane is emitted by the laser projector and intersects with the part to be measured. A perturbed laser stripe that carries the geometrical information of the profile can be captured by the camera. Since the relative position between the camera and the laser line projector is fixed, the coordinates of the points on the intersecting profile can be solved using pre-calibrated sensor parameters. As the part moves, the laser plane intersects the part at different positions and a series of intersecting profiles can be calculated. By combining these profiles with the translation distances, 3D point cloud of the part can be obtained.

The PS sensor uses the same camera and twelve spot light sources (LEDs). The light sources are arranged at equal intervals on a circular plate. Each LED is switched on/off in turn. The camera captures one image under the corresponding spot light to complete the PS measurement. The surface normal vector can be achieved according to the pre-calibrated sensor parameters, and then the depth value is calculated from the normal vector.

LSL and PS measurement are carried out sequentially. 3D measurement results from LSL are translated into the pixel coordinate system and matched with the PS results. Data interpolation of the LSL is carried out according to the pixel coordinates of the PS results so as to make the number of the two data sets consistent. The final step is to fuse the 3D point cloud of the LSL with the normal vector of the PS to achieve high precision and high clarity results.

## 3. Line Structured Light Measurement

Suppose that *P* has the camera coordinates of (*x_c_*, *y_c_*, *z_c_*) and the corresponding world coordinates of (*X_w_*, *Y_w_*, *Z_w_*), then

(1) $${\left[x_{c}\;y_{c}\;z_{c}\right]}^{T}=R{\left[X_{w}\;Y_{w}\;Z_{w}\right]}^{T}+T,$$

where **R** is the rotation matrix and **T** is the translation vector. Let *p* (*x*, *y*) be the projection point of *P* on the normalized image plane with coordinates of

(2) $$\left[x,y\right]=\left[f_{x}\cdot x_{c}/z_{c},f_{y}\cdot y_{c}/z_{c}\right],$$

The projected coordinates after considering radial and tangential distortions are

(3) $$\left\{\begin{array}{l} x^{\prime }=x(1+k_{1}r^{2}+k_{2}r^{4})+2p_{1}xy+p_{2}(r^{2}+2x^{2})\\ y^{\prime }=y(1+k_{1}r^{2}+k_{2}r^{4})+p_{1}(r^{2}+2y^{2})+2p_{2}xy \end{array}\right.,$$

where *k*_1_, *k*_2_, *p*_1_, and *p*_2_ are the distortion coefficients and *r*^2^ = *x*^2^ + *y*^2^. The pixel coordinates of *P* can be derived from Equation (4):

(4) $${\left[u\;v\;1\right]}^{T}=A{\left[x^{\prime }\;y^{\prime }\;1\right]}^{T},$$

(5) $$A=\left[\begin{array}{ccc} f_{x} & 0 & u_{0}\\ 0 & f_{y} & v_{0}\\ 0 & 0 & 1 \end{array}\right],$$

where **A** is the internal matrix, *f_x_* and *f_y_* are the focal lengths, and *u*_0_ and *v*_0_ are the coordinates of the camera principal point. Camera coordinates of the points on the laser stripe can be obtained by taking images of the planar target and the corresponding laser stripe in different positions [2]. The point cloud of the laser stripe is fitted by the random sample consensus (RANSAC) algorithm [32] to obtain a more accurate laser plane equation, as shown in Equation (6).

(6) $$B_{1}x_{c}+B_{2}y_{c}+B_{3}z_{c}+B_{4}=0,$$

where *B*_1_, *B*_2_, *B*_3_ and *B*_4_ are the coefficients of the laser plane equation. For any laser stripe image, the pixel coordinates of the stripe center are extracted using the improved gray gravity method [33]. The normalized image plane coordinates after aberration correction can be computed from Equations (3) and (4) in turn. Then, the camera coordinate of the cross-section profile is obtained by Equations (2) and (6). Motional direction is achieved by taking two images of the target at different translation positions [2].

## 4. Photometric Stereo Measurements

**Figure 2:**
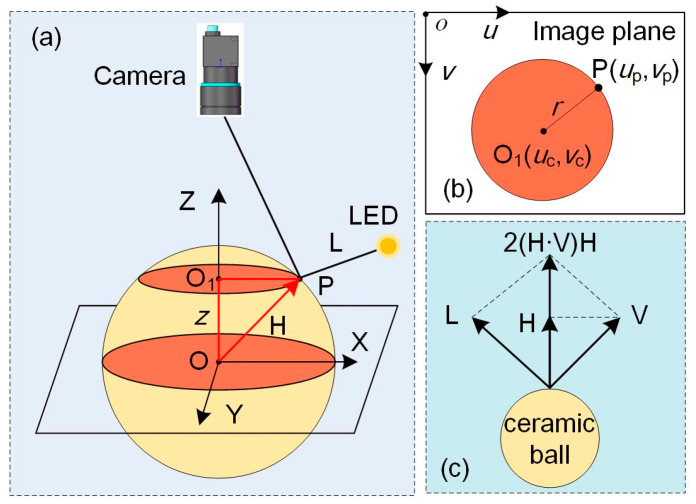
Light source direction calibration: (**a**) computing the spherical normal direction, (**b**) circular section where P locates, and (**c**) calibration of light source direction.

A ceramic ball is used to successively calibrate the direction of each spot light. Let P be the highlight point on the sphere captured by camera, and **H** be the surface normal vector at P, as shown in Figure 2a. The image of point P and the corresponding cross-section are shown in Figure 2b. O_1_ is the pixel coordinate of the sphere center, and the radius of this cross-section is *r* = ||O_1_P||. The surface normal vector at P is

(7) $$H=\left(u_{p}-u_{c},v_{p}-v_{c},\sqrt{R^{2}-r^{2}}\right),$$

where R is the radius of the ceramic sphere. The camera view direction is **V**, and then the light source direction can be obtained by Figure 2c:

(8) $$L=2\left(H\cdot V\right)H-V,$$

From the Lambert reflection model, the luminance value *I* at any point on the surface can be expressed as

(9) I=ρN·L,

where *ρ* is the reflectivity. **N** is the surface normal vector and can be expressed by

(10) $$N={\left(L^{T}L\right)}^{-1}L^{T}I/\rho ,$$

Based on the normal vectors, the gradients *q*_x_ and *q*_y_ in *x* and *y* directions can be calculated. The depth *Z* is obtained by use of the Fourier basis function method, as shown in Equation (11).

(11) $$Z=F^{-1}\left\{-j\frac{\frac{2\pi u}{N}F\left\{q_{x}\right\}+\frac{2\pi v}{M}F\left\{q_{y}\right\}}{{\left(\frac{2\pi u}{N}\right)}^{2}+{\left(\frac{2\pi v}{M}\right)}^{2}}\right\},$$

where *F* and *F*^−1^ are the two-dimensional fast Fourier transform and its inverse transform, *u* and *v* represent the frequency indexes in the row and column directions, and *M* and *N* are the number of rows and columns of the image, respectively.

## 5. Adaptive Weighted Fusion

**Figure 3:**
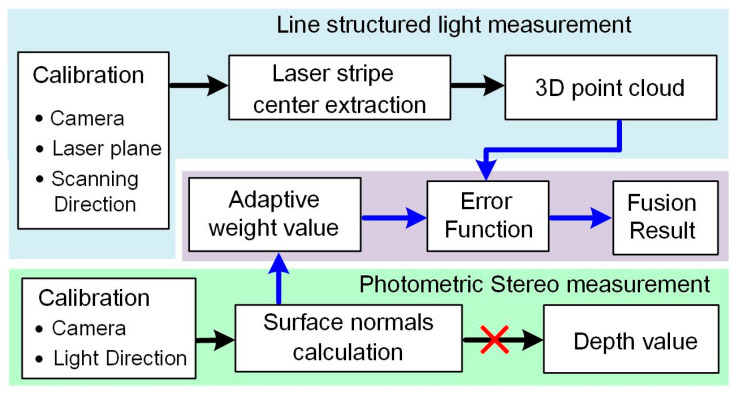
Flow chart showing the adaptive weighted fusion method.

A flowchart showing the adaptive weighted fusion method is shown in Figure 3. Firstly, the LSL and PS sensors are calibrated. Next, the 3D point cloud obtained from the LSL is fused with the normal vector obtained from the PS. The fusion is performed by minimizing an error function to obtain the optimized surface, which consists of a depth error and a surface normal vector error. Adaptive weights are calculated from the angles between adjacent normal vectors. With the method, the depth value will no longer need to be calculated from the surface normal vectors.

**Figure 4:**
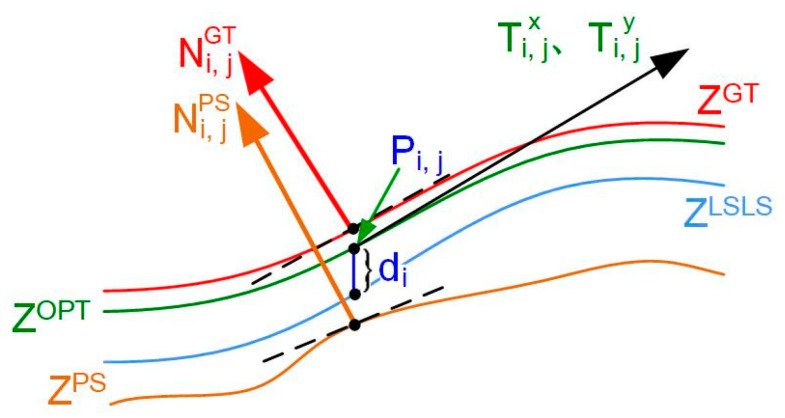
Illustration of the fusion principle.

The fusion principle is shown in Figure 4. **Z**^GT^ is the true depth, and **Z**^PS^ is the PS value. $N_{i,j}^{GT}$ and $N_{i,j}^{PS}$ are the corresponding normal vectors. **Z**^LSLS^ is the profile from LSL, **Z**^OPT^ is the optimized depth, and *P_i_*_,*j*_ represents the points at pixel position (*u*, *v*) above it; *d_i_* is the distance from *P_i_*_,*j*_ to the corresponding point of the **Z**^LSLS^ profile in the vertical direction; $T_{i,j}^{x}$ and $T_{i,j}^{y}$ are the tangent vectors of **Z**^OPT^ in the *x* and *y* directions at the pixel (*u*, *v*). With the fusion, the optimal depth value can be calculated for each pixel (*u*, *v*).

The 3D coordinates of *P_i_*_,*j*_ can then be expressed as

(12) $$P_{i,j}(u,v)={\left[-\frac{u}{f_{x}}Z_{i,j}^{\mathrm{OPT}}(u,v)-\frac{v}{f_{y}}Z_{i,j}^{\mathrm{OPT}}(u,v)Z_{i,j}^{\mathrm{OPT}}(u,v)\right]}^{T},$$

where $Z_{i,j}^{OPT}$(*u*, *v*) is the depth of the surface point at (*u*, *v*), and *f*_x_ and *f*_y_ are the camera focal lengths. Based on the error between the LSL measured profile and the optimized profile in the depth direction, the depth error function is constructed as

(13) $$E^{p}=\frac{1}{M\times N}\sum_{i=1}^{M\times N}{\left(\mu_{i,j}Z_{i,j}^{\mathrm{OPT}}-\mu_{i,j}Z_{i,j}^{\mathrm{LSLS}}\right)}^{2},$$

where *µ_i_*_,*j*_ = [−*u*/*f_x_*, −*v*/*f_y_*, 1]^T^ and $Z_{i,j}^{LSLS}$ are the depth values obtained from the LSL measurements.

**Figure 5:**
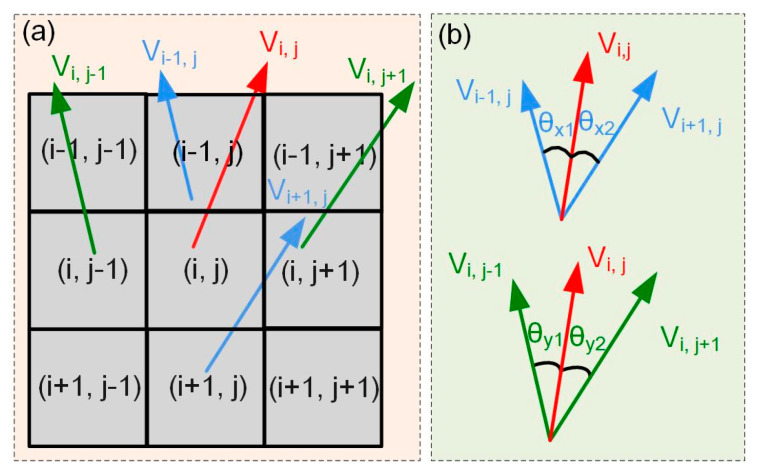
Weights computation using normal vectors: (**a**) normal vector neighborhood and (**b**) angle between adjacent normal vectors.

Normal vectors would change dramatically within the detail-rich region and slightly in the flat region. Thus, the weights of the pixel points can be assigned according to the normal vector angles between the current pixel and its neighbors. The computation principle for weights is shown in Figure 5, where Figure 5a is the neighborhood of normal vectors and Figure 5b is the angle change of the adjacent normal vectors.

Normal vector $N_{i,j}^{PS}$ at point *P_i_*_,*j*_ can be expressed as

(14) $$N_{i,j}^{\mathrm{PS}}={\left[N_{x},N_{y},N_{z}\right]}^{T},$$

Assuming that the number of rows and columns of points *P_i_*_,*j*_ in the image are *i* and *j*, respectively, then the normal vectors at points *P_i_*_,*j*_ can be represented by Equation (15).

(15) $$V_{i,j}={\left[N_{x}(i,j),N_{y}(i,j),N_{z}(i,j)\right]}^{T},$$

At this time, the normal vectors of the neighboring points on the left and right of point *P_i_*_,*j*_ are **V***_i_*_,*j*−1_ and **V***_i_*_,*j*+1_, and the normal vectors of the neighboring points on the top and bottom sides of point *P_i_*_,*j*_ are **V***_i_*_−1,*j*_ and **V***_i_*_+1,*j*_. The angle between point *P_i_*_,*j*_ and its neighboring points in the *X*-direction and the angle in the *Y*-direction can be calculated.

(16) $$\left\{\begin{array}{l} \theta_{i,j}^{x}=arc\cos\left(\frac{V_{i,j}\cdot V_{i,j-1}}{\left|V_{i,j}\right|\left|V_{i,j-1}\right|}\right)+arc\cos\left(\frac{V_{i,j}\cdot V_{i,j+1}}{\left|V_{i,j}\right|\left|V_{i,j+1}\right|}\right)\\ \theta_{i,j}^{y}=arc\cos\left(\frac{V_{i,j}\cdot V_{i-1,j}}{\left|V_{i,j}\right|\left|V_{i-1,j}\right|}\right)+arc\cos\left(\frac{V_{i,j}\cdot V_{i+1,j}}{\left|V_{i,j}\right|\left|V_{i+1,j}\right|}\right) \end{array}\right.,$$

After calculating the angles between the normal vectors at points *P_i_*_,*j*_ and their neighbors, a weight function on the magnitude of the angle between the normal vectors is obtained as

(17) $$W_{i,j}=\theta_{i,j}^{x}+\theta_{i,j}^{y},$$

At this point, the normal vectors $N_{i,j}^{\mathrm{APS}}$ after adding the weighting function are

(18) $$N_{i,j}^{\mathrm{APS}}=N_{i,j}^{\mathrm{PS}}\cdot W,$$

From Equation (12) the tangent vector at *P_i_*_,*j*_ is

(19) $$\left\{\begin{array}{l} Tx_{i,j}=\frac{\partial P_{i,j}}{\partial u}={\left[-\frac{1}{f_{x}}(u\frac{\partial Z_{i,j}^{\mathrm{OPT}}}{\partial u}+Z_{i,j}^{\mathrm{OPT}})-\frac{1}{f_{y}}v\frac{\partial Z_{i,j}^{\mathrm{OPT}}}{\partial u}\frac{\partial Z_{i,j}^{\mathrm{OPT}}}{\partial u}\right]}^{T}\\ Ty_{i,j}=\frac{\partial P_{i,j}}{\partial v}={\left[-\frac{1}{f_{x}}u\frac{\partial Z_{i,j}^{\mathrm{OPT}}}{\partial v}-\frac{1}{f_{y}}(v\frac{\partial Z_{i,j}^{\mathrm{OPT}}}{\partial v}+Z_{i,j}^{\mathrm{OPT}})\frac{\partial Z_{i,j}^{\mathrm{OPT}}}{\partial v}\right]}^{T} \end{array}\right.,$$

In ideal case, the tangent vector is perpendicular to the normal vector and its projection along the direction of the normal vector is zero. Based on the relationship between the normal vector of the PS and the tangent vector of the ideal result, the normal vector error function is constructed as

(20) $$E^{n}=\frac{1}{M\times N}\sum_{i=1}^{M\times N}\left\{{\left[Tx_{i,j}(P_{i,j})\cdot N_{i,j}^{\mathrm{APS}}\right]}^{2}+{\left[Ty_{i,j}(P_{i,j})\cdot N_{i,j}^{\mathrm{APS}}\right]}^{2}\right\},$$

Finally, by combining the depth error function of the LSL and the normal vector error function of the PS, the fusion can be achieved by minimization of the error function, and is expressed by

(21) $$Z^{\mathrm{OPT}}=\arg\underset{Z^{\mathrm{OPT}}}{\min}\left\{\lambda E^{p}+(1-\lambda )E^{n}\right\}$$

where λ ∈ [0,1], which is used to control the degree of influence of the point cloud values and normal vectors on the fused result; the smaller *λ* is, the fusion result is influenced more by the normal vectors; the larger *λ* is, the fusion result is influenced more by the 3D point cloud of the LSL.

## 6. Measurement Results and Discussions

**Figure 6:**
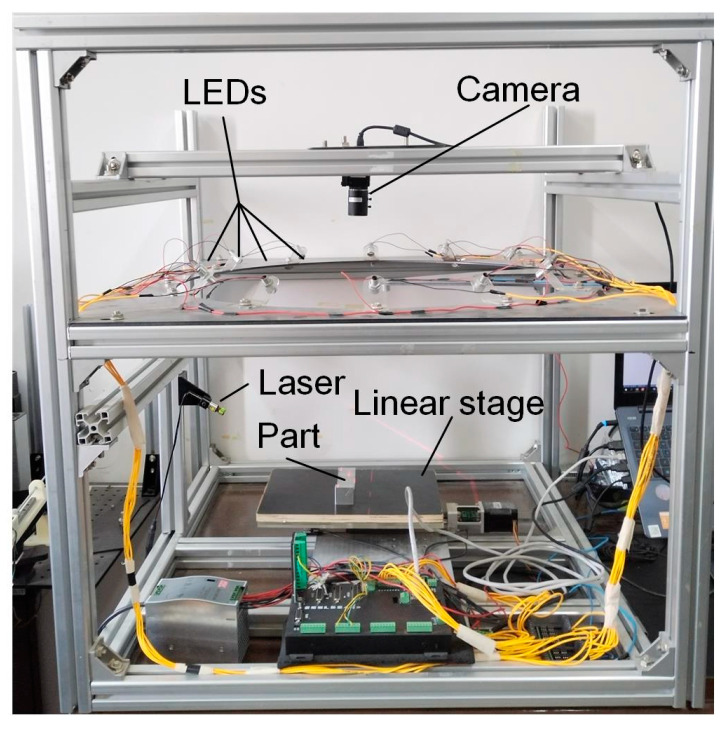
The LSL-PS cooperative measurement system.

The cooperative measurement system is shown in Figure 6. It consists of a laser line projector (Shengzuan Laser, Shenzhen, China), a camera (MV-UB500M, MindVision, Shenzhen, China), 12 LED light sources, a linear stage, and the components connecting them together. The laser line projector has a wavelength of 650 nm and a power of 5 mW. The minimum line width can reach 0.4 mm at the projection distance of 300 mm. The resolution of the camera is 800 × 600 pixels, and the focal length of the lens is 4–12 mm, which can be adjusted manually. The angle between the camera optical axis and the laser plane is about 60° and the scanning is 10 mm/s for the following experiments. The LED light sources are mounted around the camera on equally spaced circular panels. The luminance of each source is the same, and the tilt angle of the light sources and the camera’s optical axis is about 45°. The image plane of the camera is parallel to the circular plane where the light source is located. The radius of the circular plane where the light source is located is 600 mm. About 1.2 s is needed to obtain the part images under different light spots for the PS measurement. The computer has an Intel i5-8300 CPU and 4 GB RAM.

### 6.1. Measurement and Evaluation of Stairs

**Figure 7:**
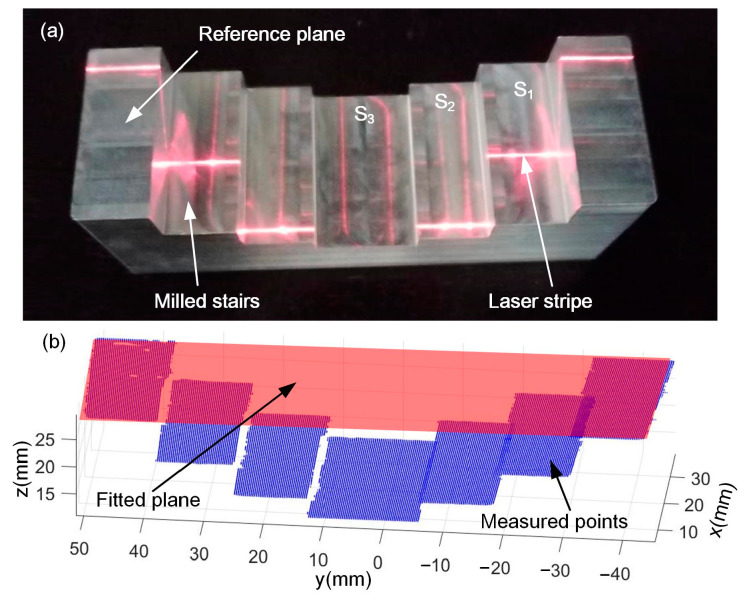
Measurement stairs using the LSL sensor: (**a**) stairs and (**b**) measured point cloud.

To verify the effectiveness of the system, measurement was carried out for precision-milled stairs, as shown in Figure 7a. The topmost step serves as the reference plane, and the remaining steps are named S_1_, S_2_, and S_3_. The heights between the steps and the reference plane are denoted by H_1_, H_2_, and H_3_. The diffused laser stripes can be seen from the steps, which are fine and bright to ensure the accuracy. Figure 7b shows the point cloud, and the boundary points on the steps are excluded before evaluation. The reference plane was first calculated by plane fitting and then the average distance from each step to the reference plane was calculated. Similarly, the heights of the steps were measured on a CMM (Hexagon GLOBAL 7107, Qingdao, China) using the topmost step as the reference plane with the measurement error less than 3 μm. Measurement results and errors are shown in Table 1. The mean absolute error (MAE) of the deviation in H_3_ is 0.0735 mm and the relative error (RE) is 0.41%, which indicates high measurement accuracy of the LSL sensor.

When the laser plane is calibrated with the RANSAC algorithm, the measurement accuracy can be further improved, as shown in Table 2. The MAE of H_3_ is 0.0328 mm and the relative error is reduced from 0.41% to 0.18%. The REs of H_1_ and H_2_ are also reduced significantly.

**Figure 8:**
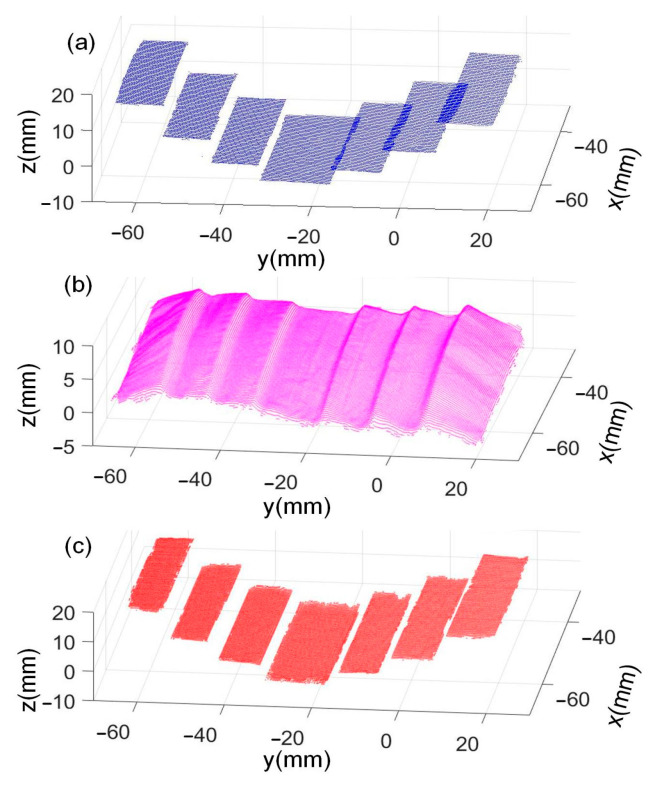
Measurement results using the different methods: (**a**) LSL, (**b**) PS, and (**c**) fused results.

Afterward, the fusion was performed and the results are shown in Figure 8. Figure 8a–c are the LSL, the PS, and the fusion results. The LSL measurement results and the fusion results are close to each other, while the PS result has a larger error because the light source in the photometric stereo measurement is not a uniform parallel light source, which leads to an error in the normal vector, and then the error accumulates in calculating the depth value, which leads to a larger overall bias in the PS measurement results.

Measurement errors of the LSL, the PS, and the fused results are evaluated by comparing with the CMM results, as shown in Table 3. It can be seen that the absolute error (AE) of the LSL measurement result of H_3_ is 0.0349 mm, the error of the PS measurement result is 0.9620 mm, and the error of the fused result is 0.0293 mm. The error of fused result is reduced by 16.0% compared to the LSL measurement result, and 97.0% compared to that of the PS measurement result. Therefore, the fusion method can further improve the accuracy.

### 6.2. Effect of Different Values of λ

**Figure 9:**
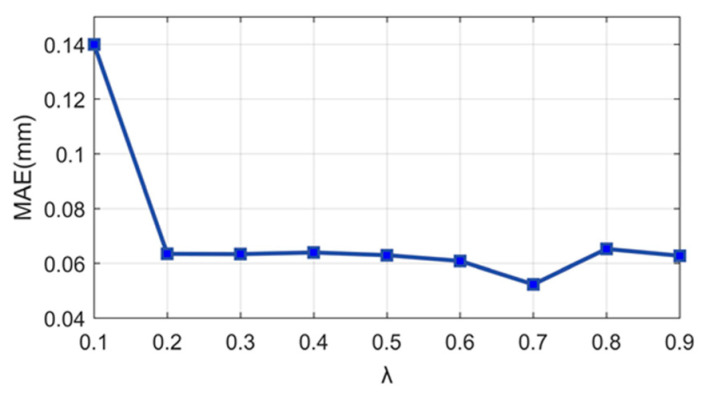
Mean absolute error for different *λ*.

Different values of *λ* were analyzed to show its impact on the fusion results, as shown in Figure 9. The sum of MAEs of the steps (H_1_, H_2_, and H_3_) varies along with *λ*. When *λ* is 0.1, the error is the largest. With a gradual increase in *λ*, the overall trend of the error value is decreasing, and when *λ* is 0.7, the error is the smallest.

**Figure 10:**
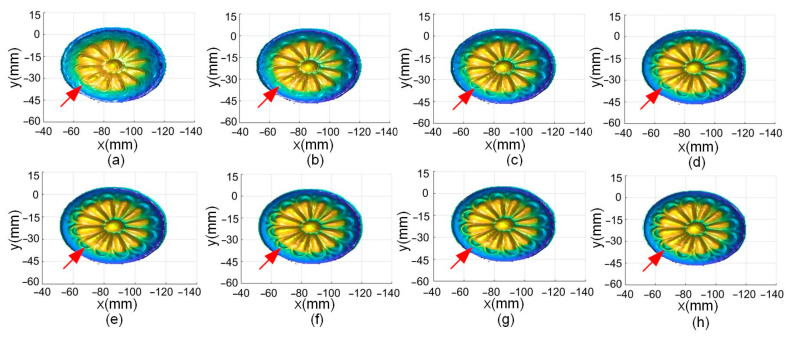
Fusion results of an aluminum part for different values of λ: (**a**) λ = 0.1, (**b**) λ = 0.2, (**c**) λ = 0.3, (**d**) λ = 0.4, (**e**) λ = 0.5, (**f**) λ = 0.6, (**g**) λ = 0.7, and (**h**) λ = 0.8.

The effect on the clarity of the fusion result when taking different *λ* was also analyzed. Measurement results of an aluminum part are fused, and the results are shown in Figure 10.

For Figure 10a–f, the same position is analyzed that is at the outermost edge of the petal indicated by the arrow. When *λ* = 0.1 and 0.2, it can be seen that the details in this region are relatively blurred. When *λ* = 0.3, the details become somewhat clearer. When *λ* = 0.4, the undulations at edge of the petals in this region are further increased, which is more closely matched with the actual object. In addition, a small bump starts to appear at the upper left of the arrow. In Figure 10f–h, the small bumps are no longer changing compared to Figure 10e. Therefore, when *λ* is greater than 0.5, the clarity of the fusion result has stabilized. Combining the results of the accuracy at different values of *λ*, *λ* is taken as 0.7 when fusion is performed.

### 6.3. Measurement of Complex Parts

**Figure 11:**
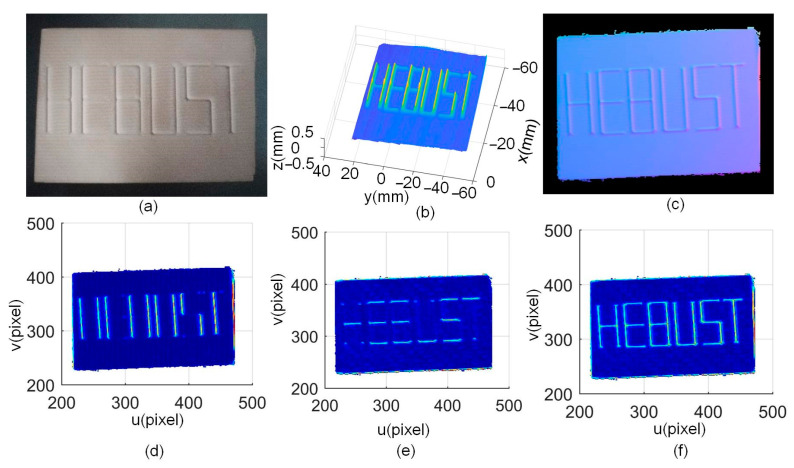
Measurement of a machined part with letters: (**a**) the part, (**b**) LSL measurement results, (**c**) PS normal vectors, (**d**) angle of the adjacent normal vector in the X direction, (**e**) angle of the adjacent normal vector in the Y direction, and (**f**) fused results.

The purpose of this measurement system is to obtain 3D geometric information of complex parts, which can be used for quality inspection and reverse engineering. Firstly, six letters ”HEBUST“ were milled by a precision machine. Figure 11a shows the machined parts. The measurement result using the LSL sensor is shown in Figure 11b. The normal vector calculated using PS is shown in Figure 11c, where each letter can be seen. The angle of the normal vector in the *X* and *Y* directions is calculated using the proposed method, as shown in Figure 11d and e, respectively. The letters can only be clearly observed in the corresponding directions. Figure 11f is the fused result where each letter becomes very clear, the same as that of Figure 11c. The running time for the fusion is about 8 s.

**Figure 12:**
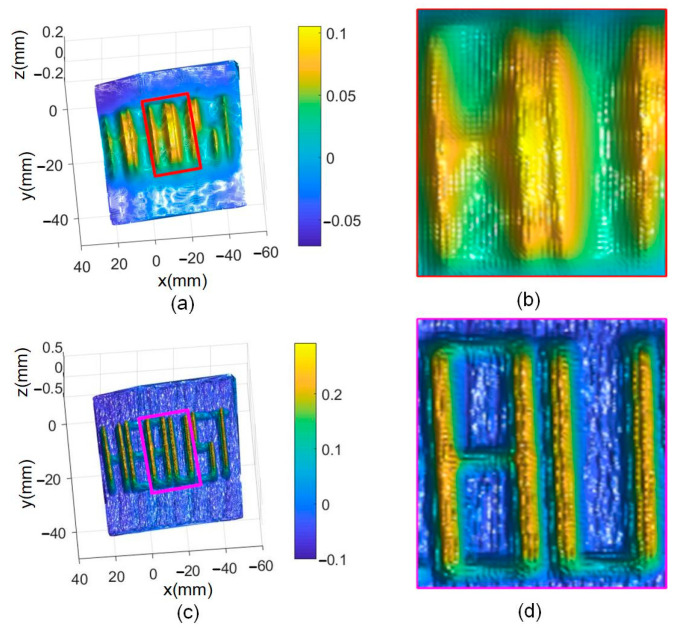
Fusion results of “HEBUST” and its details: (**a**) Nehab method, (**b**) enlargement of Nehab method, (**c**) our method, and (**d**) enlargement of our method.

The fusion results of “HEBUST” are shown in Figure 12. The fused result of the “HEBUST” by Nehab method [20] is shown in Figure 12a, where the six letters can be seen, but the lateral part of the letters is insufficiently clear. In contrast, with the proposed method all of the letters can be seen clearly, as shown in Figure 12c. Figure 12b,d are the enlargements denoted in Figure 12a,c, respectively. Note that the features of the two letters “BU” in the transverse direction are very fuzzy in Figure 12b. When using our method, these letters become very clear and the transverse features can be seen.

**Figure 13:**
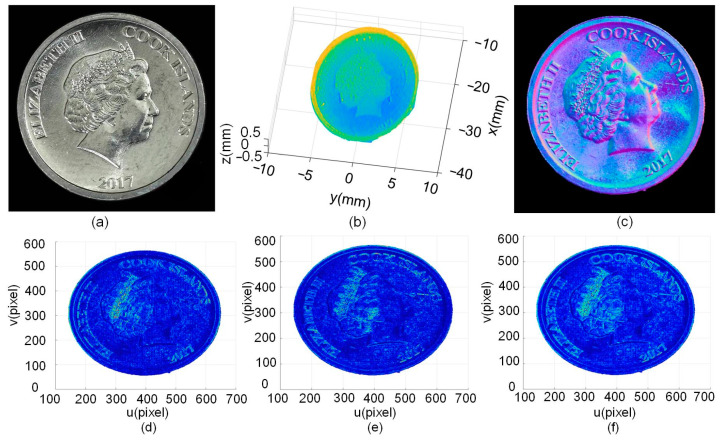
Measurement of coin parts: (**a**) coin, (**b**) LSL sensor measurement results, (**c**) PS normal vectors, (**d**) angle of adjacent normal vector in the *X* direction, (**e**) angle of adjacent normal vector in the *Y* direction, and (**f**) fused results.

To further verify the effectiveness of the proposed method, a coin with rich texture information was measured. These textures include portraits, letters, and numbers. Figure 13b is the measurement result of the LSL where the approximate outline can be seen, but the details are not clear. Figure 13c shows the normal vector calculated from the PS, which clearly shows its detailed features. The angles of the normal vector in *X* and *Y* directions are calculated using the proposed method, as shown in Figure 13d,e, respectively. The coin can only be clearly characterized in the corresponding directions. Figure 13f is the fused result where detailed features such as the characters, letters, and numbers on the coin become clear.

**Figure 14:**
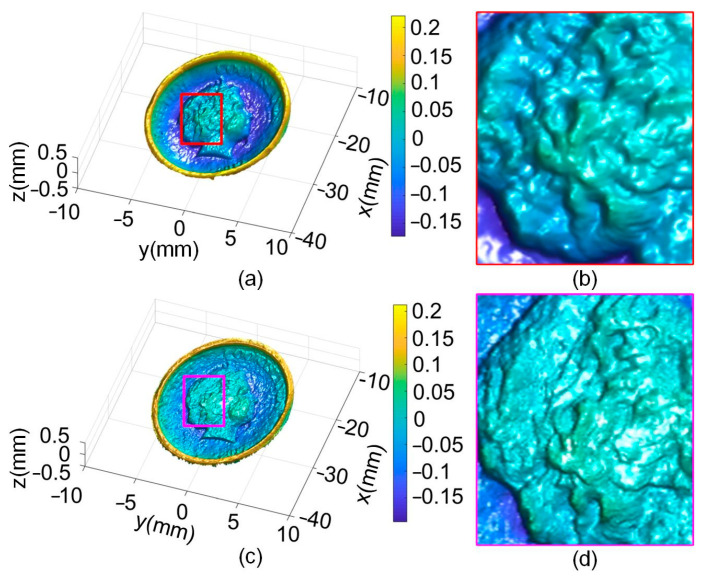
Fusion result of the coin: (**a**) Nehab method, (**b**) enlargement of (**a**), (**c**) our method, and (**d**) enlargement of (**c**).

The fusion result of the coin is shown in Figure 14. Figure 14a shows the detail achieved by the Nehab method. The fusion result using the proposed method is shown in Figure 14c. Computing time required for data fusion was about 6 s. The details of the result in the middle position are clearer compared to the Nehab method. Enlargement of the fusion result is also shown. In Figure 14b, the approximate features of the hair can be observed, but it is insufficiently clear. In Figure 14d, it becomes very clear with our method.

**Figure 15:**
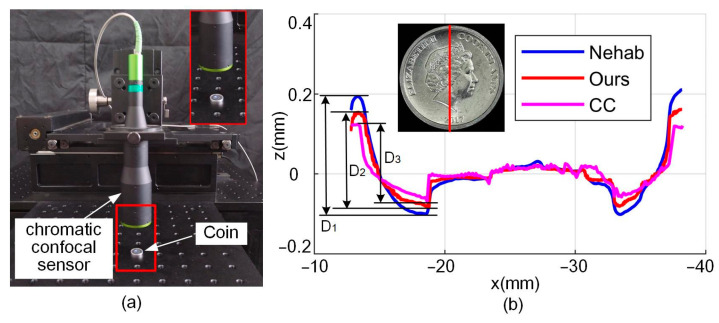
Comparison of fusion results for coins: (**a**) chromatic confocal sensor and (**b**) cross-section profile obtained using the different methods.

A cross-section profile of the coin is selected for comparative analysis, as shown in Figure 15. This profile was obtained by use of the Nehab method, the proposed method, and a chromatic confocal (CC) sensor, respectively. The CC sensor (Liyi D35A18R8S25, Shenzhen, China) is shown in Figure 15a, with a resolution of 40 nm and a linear accuracy of up to ±2 µm. Measurement accuracy of the CC sensor is very high, so it can be used as the reference for accuracy evaluation of the fused results.

Figure 15b shows the measurement result of the center profile; it can be seen that the peak to valley value of the profile is D_1_ = 0.2598 mm using the Nehab method. With our method D_2_ = 0.2334 mm, and the reference value D_3_ is 0.1901 mm. The deviation between the Nehab method and the CC sensor is 0.0697 mm. With the proposed method, the deviation is reduced to 0.0433 mm, a reduction of 37.9%. Therefore, the proposed method not only improves the clarity, but also improves the accuracy.

## 7. Conclusions

A LSL-PS cooperative measurement system is designed, and an adaptive weighted data fusion method is proposed. The adaptive fusion is based on the normal vector that is computed with the PS method. The 3D point cloud obtained from the LSL can be directly fused with the normal vector from the PS. Therefore, the integration process can be eliminated in the PS measurement, which avoids the error accumulation. The weight function based on the angle of the normal vector is added to the normal vector error function, which makes the features of the fusion result clearer. More experiments will be carried out in the future for complex surfaces with fine features.

## Figures and Tables

**Table 1:** Measurement results for the stairs (unit: mm).

No.	CMM	1	2	3	4	5	MAE	RE
H_1_	7.9992	7.9664	7.9703	7.9895	7.9734	7.9741	0.0245	0.31%
H_2_	13.9982	13.9340	13.9270	13.9491	13.9377	13.9331	0.0620	0.44%
H_3_	17.9987	17.9987	17.9206	17.9167	17.9421	17.9208	0.0735	0.41%

**Table 2:** Measured results for aluminum stairs using the RANSAC (unit: mm).

No.	CMM	1	2	3	4	5	MAE	RE
H_1_	7.9992	8.0009	7.9846	8.0018	7.9862	8.0023	0.0040	0.05%
H_2_	13.9982	13.9694	13.9664	13.9797	13.9594	13.9809	0.0270	0.19%
H_3_	17.9987	17.9680	17.9607	17.9702	17.9499	17.9809	0.0328	0.18%

**Table 3:** Comparison of measured results using different methods (unit: mm).

No.	CMM	LSL	AE	PS	MAE	Fusion	AE
H_1_	7.9992	7.9791	0.0201	6.1526	1.8466	8.0095	0.0103
H_2_	13.9982	13.9919	0.0063	11.3644	2.6338	14.0121	0.0139
H_3_	17.9987	18.0336	0.0349	17.0367	0.9620	18.0280	0.0293

## Data Availability

The original contributions presented in the study are included in the article, further inquiries can be directed to the corresponding author.
